# Supplementary material for: Reconciling Mining with the Conservation of Cave Biodiversity: A Quantitative Baseline to Help Establish Conservation Priorities
Source: PLoS One. 2016 Dec 20;11(12):e0168348. doi: 10.1371/journal.pone.0168348 (PMC5173368; doi:10.1371/journal.pone.0168348)
Supplement: S1 Dataset — (ZIP) [file pone.0168348.s002.zip › Taxa/Serra Norte/SN_2013/N5S/Lista N5S-09.pdf]

CAVIDADE N5S-0009

Classe	Ordem	Fam/Outros	Gên/Outros	Espécie	S-U	U-S	Zon.
Annelida	Oligochaeta			jovem	4		P/A
Arachnida	Acari	Cunaxidae		sp.1	X		P
Arachnida	Acari	Eupodidae		sp.1	X		E/P/A
Arachnida	Acari	Laelapidae	*Cosmolaelaps*	sp.1	X		P
Arachnida	Acari	Laelapidae		sp.3	X		A
Arachnida	Acari	Rhagidiidae		sp.20	X		P
Arachnida	Acari	Trombidiformes		sp.49	X		P/A
Arachnida	Acari			jovem		X	
Arachnida	Amblypygi	Charinidae	*Charinus*	sp.1	1		E
Arachnida	Amblypygi	Phrynidae	*Heterophrynus*	*longicornis*		X	
Arachnida	Araneae	Barychelidae		jovem	1		E
Arachnida	Araneae	Caponiidae		sp.1	X	X	E/P
Arachnida	Araneae	Corinnidae	*Myrmecium*	sp.1	1		P
Arachnida	Araneae	Ctenidae	*Isoctenus*	sp.1	1	1	
Arachnida	Araneae	Drymusidae	*Drymusa*	*spelunca*	X		P
Arachnida	Araneae	Ochyroceratidae	*Ochyrocera*	sp.1	X	X	P/A
Arachnida	Araneae	Oonopidae		jovem	X		P
Arachnida	Araneae	Pholcidae	*Leptopholcus*	sp.1	X		E
Arachnida	Araneae	Theridiidae	*Anelosimus*	sp.1	X		P
Arachnida	Araneae	Theridiidae	*Episinus*	gr. *cognatus*	X		E
Arachnida	Araneae	Theridiidae		jovem		X	
Arachnida	Araneae	Theridiosomatidae	*Plato*	sp.1	X	X	E/P
Arachnida	Opiliones	Escadabiidae		sp.1	X		E
Arachnida	Opiliones	Escadabiidae		sp.2	X	X	E/P
Arachnida	Opiliones	Gonyleptidae	Tricommatinae	sp.1	X		E
Arachnida	Opiliones	Minuidae		jovem		X	
Arachnida	Palpigradi	Eukoeneniidae	*Allokoenenia*	sp.1	X		E
Arachnida	Palpigradi	Eukoeneniidae	*Eukoenenia*	sp.1	X		E
Arachnida	Palpigradi			jovem	X	X	P
Arachnida	Pseudoscorpiones	Bochicidae		jovem		X	
Arachnida	Pseudoscorpiones	Chthoniidae	*Pseudochthonius*	sp.2	X		P
Chilopoda	Geophilomorpha	Geophilidae		sp.3	1		P
Chilopoda	Geophilomorpha	Schendylidae		sp.1	5		P
Chilopoda	Scolopendromorpha	Cryptopidae	*Cryptops*	sp.1	2		E
Chilopoda	Scolopendromorpha	Scolopendridae	*Rhoda*	sp.1	1		P
Chilopoda	Scolopendromorpha	Scolopocryptopidae	*Dinocryptops*	jovem	1		A
Chilopoda	Scolopendromorpha	Scolopocryptopidae	*Newportia*	sp.1	1		E
Chilopoda	Scutigeromorpha	Pselliodidae	*Sphendononema*	jovem	5		E/P
Diplopoda	Polydesmida	Chelodesmidae		sp.3	1		P
Diplopoda	Polydesmida	Pyrgodesmidae		sp.1	X	X	P/A
Entognatha	Collembola	Entomobryidae		sp.4	X		P
Entognatha	Collembola	Isotomidae		sp.1	X		E
Entognatha	Collembola	Paronellidae		sp.1	X		P
Entognatha	Collembola	Paronellidae		sp.4	X		P
Entognatha	Collembola	Sminthuroidea		sp.2	X	X	P
Entognatha	Diplura	Campodeidae		sp.1	X		E/P
Entognatha	Diplura	Japygidae		sp.3	X	X	P
Entognatha	Diplura	Projapygidae		sp.1	X		P
Insecta	Blattodea	Blattellidae		sp.1	4		P

Insecta	Coleoptera	Carabidae	*Coarazuphium*	sp.n.1	X		P
Insecta	Coleoptera	Carabidae		sp.11	X	X	P
Insecta	Coleoptera	Chrysomelidae		sp.4	X		P
Insecta	Coleoptera	Dytiscidae		sp.11C	X		P
Insecta	Coleoptera	Dytiscidae		sp.22	X		P
Insecta	Coleoptera	Erotylidae		sp.1	X		E
Insecta	Coleoptera	Staphylinidae	Pselaphinae	sp.3	X		P
Insecta	Coleoptera	Staphylinidae	Pselaphinae	sp.12	X		P
Insecta	Coleoptera	Staphylinidae		sp.4	X		P
Insecta	Diptera	Conopidae	*Stylogaster*	sp.	X		P
Insecta	Diptera	Culicidae	*Culex*	sp.	X		P/A
Insecta	Diptera	Phoridae	Phorinae	sp.	X		P
Insecta	Diptera	Psychodidae	Phlebotominae	sp.	X		E/P
Insecta	Heteroptera	Dipsocoroidea		sp.	X		P
Insecta	Heteroptera	Nabidae		sp.	X		E
Insecta	Heteroptera	Reduviidae	Reduviinae	sp.	1		E
Insecta	Heteroptera	Veliidae	*Paravelia*	sp.	X	X	P
Insecta	Homoptera	Cixiidae		jovem	X	X	E/P
Insecta	Hymenoptera	Formicidae	*Ectatomma*	sp.1	X		E
Insecta	Hymenoptera	Formicidae	*Paratrechina*	sp.1	X		E/P
Insecta	Hymenoptera	Formicidae	*Paratrechina*	sp.2	X		P
Insecta	Hymenoptera	Formicidae	*Pheidole*	sp.1	X		P
Insecta	Hymenoptera	Formicidae	*Solenopsis*	sp.1	X	X	E
Insecta	Isoptera	Termitidae	*Embiratermes*	sp.	X		E
Insecta	Isoptera	Termitidae	*Subulitermes*	sp.	X		E
Insecta	Lepidoptera	Tineoidea		sp.1	X	X	E
Insecta	Orthoptera	Phalangopsidae	*Paraclodes*	sp.	3		E
Insecta	Orthoptera	Phalangopsidae	*Phalangopsis*	sp.	24		E
Insecta	Psocoptera			jovem	X		E/P
Malacostraca	Isopoda	Scleropactidae	*Circoniscus*	*buckupi*	X		P/A
Malacostraca	Isopoda	Scleropactidae		sp.		X	
Mammalia	Chiroptera	Emballonuridae	*Peropteryx*	*kappleri*	2		
Mammalia	Chiroptera	Furipteridae	*Furipterus*	*horrens*	1		
